# Distribution and frequency of genetic mutations in three insecticide targets in field populations of *Culex tritaeniorhynchus* in Mianyang City, Sichuan Province, China

**DOI:** 10.3389/fcimb.2025.1496849

**Published:** 2025-02-06

**Authors:** Hongwei Xie, Meilin Tang, Hongying Sun, Zhengzheng Huang, Mengmeng Dong, Xianying Wen

**Affiliations:** Mianyang City Center for Disease Control and Prevention, Public Health surveillance Institute, Mianyang, Sichuan, China

**Keywords:** insecticide resistance, *Culex tritaeniorhynchus*, voltage-gated sodium channel, GABA receptor Rdl subunit, acetylcholinesterase, Rdl A296S mutation

## Abstract

Japanese encephalitis (JE) is an important mosquito borne infectious disease which is mainly transmitted by *Culex tritaeniorhynchus* Giles (1901) in China. At present, vector control remains an important means to prevent and control vector-borne diseases including JE. The development of insecticide resistance has seriously threatened the effectiveness of insecticide-based vector control programmes. Therefore, understanding insecticide resistance in the target pest is essential to inform evidence-based vector control. In Mianyang City of Sichuan Province of China, *Cx. tritaeniorhynchus* is the dominant mosquito species, and JE has been documented in this city. Unfortunately, there is little information on the status and underlying mechanisms of insecticide resistance in field populations of *Cx. tritaeniorhynchus*, the main JE vector in this region. In the study, a total of 314 adults of *Cx. tritaeniorhynchus*, collected from 6 sites across Mianyang City, were inspected for resistance-conferring mutations in three genes encoding voltage-gated sodium channel (VGSC), Gamma-aminobutyric acid (GABA) receptor Rdl subunit (Rdl), and acetylcholinesterase (AChE) by DNA Sanger sequencing. The results showed that the classical L1014F mutation in VGSC was distributed in all the 6 populations at varying frequencies from 16.98% to 27.78%, and the frequency of F455W mutation in AChE was extremely high (97.06%-100%). Notably, the conserved mutations A296S and V327I previously reported in the Rdl of some other species of mosquitoes were discovered in *Cx. tritaeniorhynchus* for the first time. The frequency of the resistant Rdl 296S allele was 62.04% to 94.00%, while the V327I mutation was present at a much lower frequency ranging from 0.93% to 1.8%. Overall, the prevalent co-existence of resistance-conferring mutations in multiple insecticide target proteins in *Cx. tritaeniorhynchus* populations in Mianyang City indicates a worrying status of insecticide resistance, and suggests that it is highly required to monitor the phenotypic resistance of *Cx. tritaeniorhynchus* on a regular basis.

## 1 Introduction

Japanese encephalitis (JE) is a mosquito-borne zoonotic disease caused by Japanese encephalitis virus (JEV) (Dong et al., 2023). JE is mainly harmful to children and adolescents, causing central nervous system lesions (Kumar, 2020). The mortality rate caused by JE is about 10% - 30%, and up to 50% of survivors are left neurological defects (Campbell et al., 2011; Tolsa-Garcia et al., 2023). According to WHO statistics (https://www.who.int/data/collections), there are about 68,000 clinical cases of JE each year, with more than 3 billion people at risk of infection. Mainland China used to be a high-epidemic area for JE, but the incidence of JE has declined rapidly since the end of the 20th century (Tong et al., 2023; Yan et al., 2024). However, in recent year, JE outbreaks have been reported in central and western regions of China including Sichuan Province (Shi et al., 2022).

Currently, the main measures to control the epidemic of JE include vaccination and mosquito control. Although vaccination is the most economical and reliable measure for JE control in China, mosquito control remains an important means to prevent and control JE due to the change of the dominant JEV genotype and incomplete vaccination coverage in China (Cao et al., 2016; Dong et al., 2023; Yan et al., 2024), especially when the outbreak of JE.

While more than 20 species of mosquitoes are possible vector of JEV in China, *Culex tritaeniorhynchus* is the most important one (Yan et al., 2024). The breeding areas of *Cx. tritaeniorhynchus* are mostly rice fields, which are heavily exposed to agricultural insecticides. Due to the continuous use of insecticides in agriculture and the short generation and high fertility of *Cx. tritaeniorhynchus*, field populations of this mosquito species have developed resistance to organophosphate (OP), carbamate (CB) and pyrethroid insecticides in different regions of China (Wu et al., 2016; Shi et al., 2019; Wu et al., 2021). For example, *Cx. tritaeniorhynchus* collected from four different provinces of China were resistant to deltamethrin, beta-cypermethrin, permethrin, dichlorvos, and propoxur (Wu et al., 2016).

Two major mechanisms of insecticide resistance have been documented in mosquitoes, i.e. metabolic resistance and target resistance (Bandibabone et al., 2021; Li et al., 2021; Wang et al., 2023). Metabolic resistance is caused by enhanced insecticide detoxification, and target resistance is resulted from reduced sensitivity of insecticidal targets including acetylcholinesterase, nerve axon sodium ion channel, and γ-aminobutyric acid receptor chloride ion channel. As the target of organochlorine and pyrethroid insecticides, the voltage-gated sodium channel (VGSC) can regulate the sodium ions of cardiomyocytes, neurons and other cells, depolarization and repolarization process, and determine the state of excitable cells (Orjuela et al., 2019). Insensitivity to dichloro-diphenyl-trichloroethane (DDT) and pyrethroids caused by sodium channel point mutations is called knockdown resistance (*kdr*) (Roca-Acevedo et al., 2023). The first reported *kdr* mutation is a change of Leu to Phe at site 1014 of the *Musca domestica* sodium channel gene (*VGSC*) (Rinkevich et al., 2012). Acetylcholinesterase (AChE), as a key enzyme in biological nerve conduction, is the target site of OP and CB insecticides (Vale and Lotti, 2015). Several mutations of AChE related to insecticide resistance have been reported in mosquitoes (reviewed in Liu, 2015), including the F455W substitution identified in *Cx. tritaeniorhynchus* (Nabeshima et al., 2004). Cyclopentadiene insecticides (e.g. lindane and dieldrin) and other chemicals (e.g. fipronil) act on Gamma-aminobutyric acid (GABA) receptor in insects (Chebib et al., 2001; Chen et al., 2006; Nakao, 2017). A point mutation leading to a substitution of Ala 296 to Ser/Gly (A296S/G) in the GABA receptor Rdl subuint (Rdl) was reported to be responsible for dieldrin resistance in several species of mosquitoes (Liu, 2015).

Mianyang City is located in the northwest of Sichuan Basin of China within the north subtropical mountainous humid monsoon climate zone. The climate in Mianyang is mild throughout the year and rainfall is abundant. These natural and social factors are suitable for the spread of mosquito-borne infectious diseases. As the global climate warms and population mobility increases, Mianyang City is facing an increasing threat of these mosquito-borne diseases.

Given that resistance will undermine the effectiveness of current insecticide based JE vector control interventions, it is of great significance to carry out regular monitoring of vector resistance to commonly used chemical insecticides. Unfortunately, except a recent study on genetic mutations in populations of *Cx.tritaeniorhynchus* in southeast of Sichuan Basin of China (Liu et al., 2023), there is no other report on the status of pesticide resistance in *Cx. tritaeniorhynchu* field populations in Sichuan Province of China to our knowledge. In this study, as the first effort in this regard, we investigated the possible presence of the resistance conferring mutations in three genes encoding insecticide targets in six representative *Cx. tritaeniorhynchoides* populations in Mianyang City of Sichuan Province.

## 2 Methods

### 2.1 Samples

**Figure 1:**
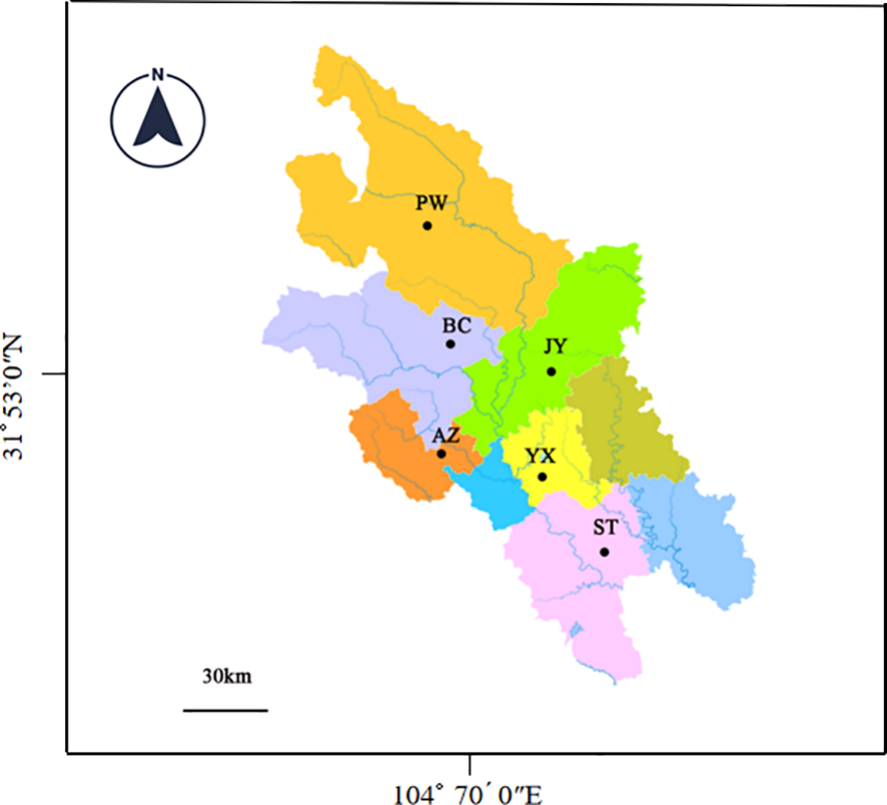
Sampling locations in the Mianyang City, Sichuan Province of China.

The sampling sites are located in Anzhou (AZ), Beichuan (BC), Jiangyou (YJ), Pingwu (PW), Santai (ST) and Youxian (YX) of Mianyang City as shown in **Figure 1**. Around these sites, rice is the main crop and *Cx. tritaeniorhynchus* is one of the dominant mosquito species. In these rice fields, pyrethroid and organophosphate insecticides have been commonly used.

The adults of *Cx. tritaeniorhynchus* were captured using 1-3 mosquito light traps around pig houses or cattle sheds during 20:00 to 8:00 at each site for 1 to 4 days from August to September in 2023. The specimens were morphologically identified and individually stored in 75% or 95% ethanol at 4°C. The species identification of samples used in this study was confirmed molecularly based on the sequences of mitochondrial COI gene.

### 2.2 Genomic DNA extraction

The genomic DNA of individual mosquitoes was isolated according to the protocol described in Rinkevich et al. (2006) with a few modifications. Briefly, individual adults were washed with sterile water for several times. The head and thorax were placed in a 1.5 mL EP tube with 50 µL of lysis buffer (100mM Tris-Hcl, pH8.0, 10 mM EDTA, 50 mM NaCl and 1% SDS), ground using TGrinder OSE-Y30 (Tiangen, China), and then supplemented with additional 300 µL of lysis buffer. 5 µL protease K (20 mg/mL) was added into the tube, and the mixture was mixed and incubated at 60°C for 1 hour. After that, 40 µL of 8 M potassium acetate solution was added into the tube, and leaved on ice for 10 min. After the mixture was centrifuged 14000 g for 30 min, 320 µL of supernatant was taken to a new centrifuge tube. Then, 640 µL of chilled anhydrous ethanol were added into the supernatant, and the samples were kept at room temperature for 20 min after mixing, followed by centrifugation at 14000 g for 20 min. The supernatant was discarded, then pellet was resuspected using 600 µL of 70% ethanol, and centrifuged at 8000 g for 15 min. The supernatant was discarded, and pellet was air-dried and dissolved in ~30 µL of sterile water. The DNA samples were stored at 4°C or -20°C.

### 2.3 PCR amplification of a fragment of the *VGSC*, *Rdl* and *Ace* gene

Primer pairs TRI5 (CTTCACCGACTTCATGCACTC, Wu et al., 2016) and Ct-kdr-R1 (TAGAAATATTGTAACCACACTGAAC, this study), Rdl-F (CAGTTTGTACGTTCGATGGGT) and Rdl-R (GGCAAATACCATGACGAAGCA), and Ct-Ace-F (GTCTAGCCGAAGCCGTCAA) and Ct-Ace-R (TTGGG ATTGCCGGTTTTGGCAAAA) were used to amplify a target-site-containing fragment of the *VGSC*, *Rdl* and *Ace* gene respectively. The PCR mixture (20 µL) consisted of 10 µL of 2×Es Taq MasterMix (CWBIO, Beijing, China), 0.5 µL of each primer (10 µM), and 1 µL of genomic DNA (50-200 ng). The PCR was run as: 94°C for 2 min, 35 cycles of 94°C for 30s, 56°C (for VGSC), or 58°C (for Rdl) for 30 s, 72°C for 15 s, and 72°C for 2 min. For Ace, the PCR mixture included 10 µL of 2×EZ3 Mix (Dakewe Biotech Co., Ltd, China), 0.75 µL of each primer, and 1 µL of genomic DNA (50-200 ng). The PCR procedure was as follows: 95°C for 3 min, 35 cycles (95°C for 10 s, 55°C for 15 s, 72°C for 30 s), 72°C for 5 min.

### 2.4 DNA sequencing and gene sequence analysis

PCR products were detected by agarose gel electrophoresis and Sanger sequenced after purification by BGI Company (Beijing, China). DNA sequences obtained by Sanger sequencing were manually checked and both-end trimmed. All confirmed sequences were aligned by Muscle 3.8 (Edgar, 2004) to identify polymorphic sites. Haplotypes were identified by directly reading from homozygotes. Basic population genetic analysis was conducted using the online software Genepop 4.7.5 (https://genepop.curtin.edu.au/).

## 3 Results

### 3.1 Distribution and frequency of the VGSC L1014F mutation

**Figure 2:**
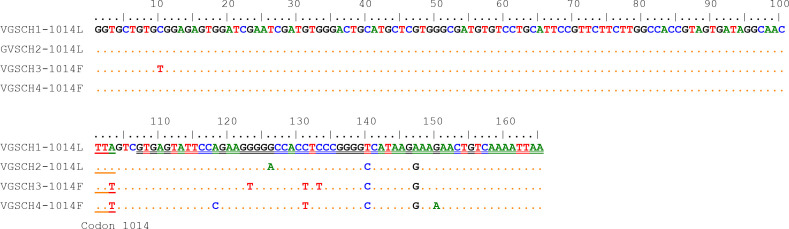
Nucleotide sequence alignment of the four *VGSC* haplotypes of the *Cx. tritaeniorhynchus* identified in this study. The codon 1014 and the intron region are indicated by single underline and double underline respectively.

Ten nucleotide polymorphic sites were identified from the 165-bp sequences of the *VGSC* gene, with 2 polymorphic sites existing in the exon region and 8 polymorphic sites in the intron region. The only nonsynonymous variation (A to T) was the nucleotide 103 that led to the amino acid replacement (Leu to Phe) corresponding to the insecticide-related amino acid residue 1014 of the VGSC. Four different haplotypes (H1 to H4), including two wild types and two resistant types, were identified from homozygotes in our samples (**Figure 2**).

**Figure 3:**
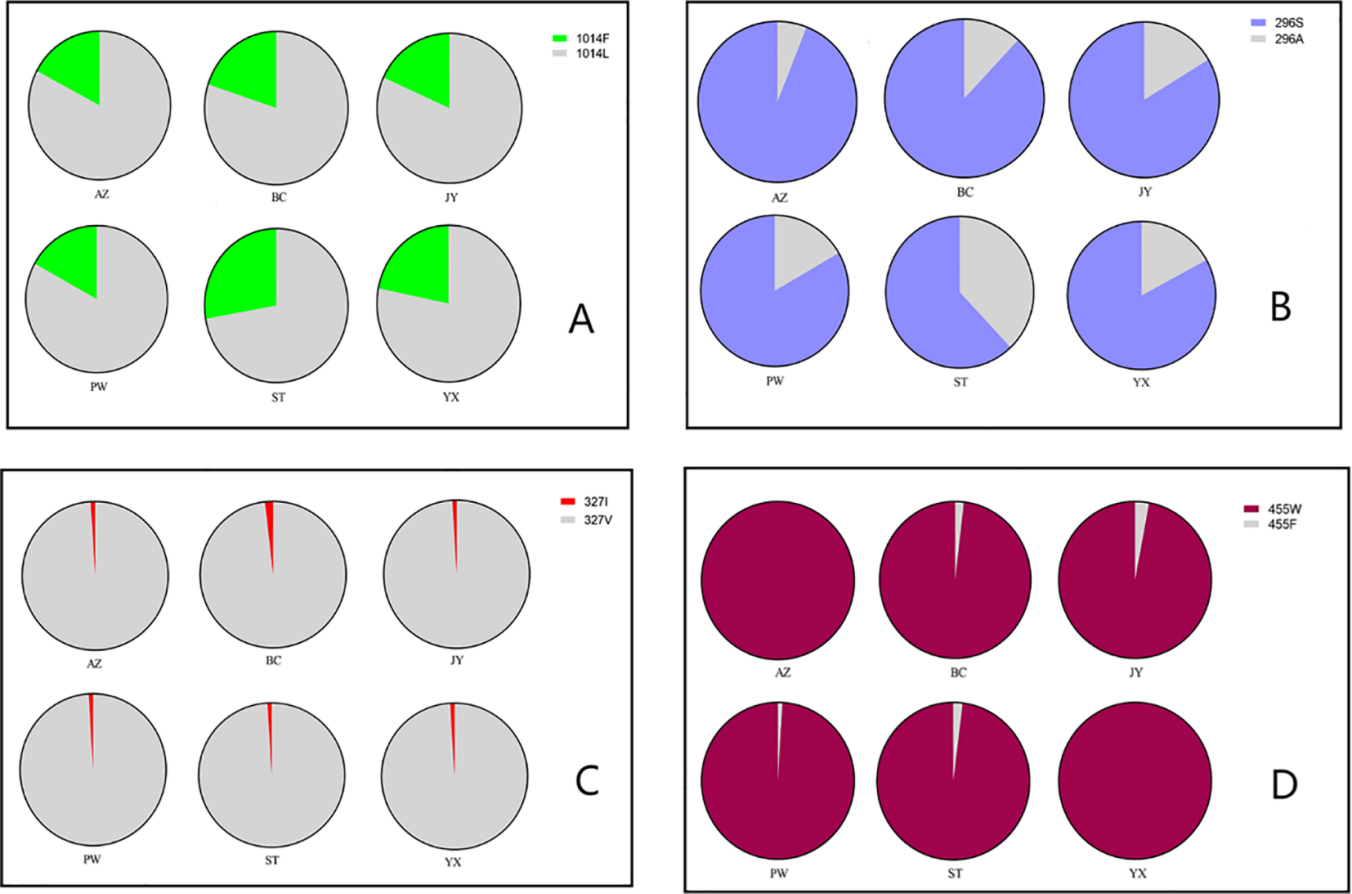
Distribution and frequency of alleles in six populations of the *Cx. tritaeniorhynchus* in Mianyang City, Sichuan Province of China. **(A)** *VGSC*-1014; **(B)** *Rdl*-296; **(C)** *Rdl*-327; **(D)** *Ace*-455.

At position 1014 of the VGSC (VGSC-1014), all three possible genotypes were detected, namely wild homozygote (TTA), resistant heterozygote (TTA/TTT), and resistant homozygote (TTT) (**Table 1**). The frequency of resistant homozygotes ranged from 0 (BC) to 6.25% (YX). Overall, all populations did not deviate from HWE (probability test, *P*>0.05), and there was no significant difference in genotype frequency among populations (exact G test, *P*=0.2913). The frequency of resistant allele (1014F) was in the range between 16.98% (PW) and 27.78% (ST) (**Figure 3A**).

**Table 1:** Frequency of individual genotypes of the *VGSC* gene at position 1014 in six *Cx. tritaeniorhynchus* populations in Mianyang City, Sichuan Province of China.

Locations	N	Genotypes		
		TTA	TTA/TTT	TTT
AZ	52	35 (67.31%)	16 (30.77%)	1 (1.90%)
BC	55	33 (60.00%)	22 (40.00%)	0 (0.00%)
JY	52	35 (67.31%)	15 (28.85%)	2 (3.85%)
PW	53	37 (69.81%)	14 (26.42%)	2 (3.77%)
ST	54	27 (50.00%)	24 (44.44%)	3 (5.56%)
YX	48	30 (62.50%)	15 (31.25%)	3 (6.25%)
Total	314	197 (62.70%)	106 (33.76%)	11 (3.5%)

### 3.2 Distribution and frequency of the Rdl A296S and V327I mutations

**Figure 4:**
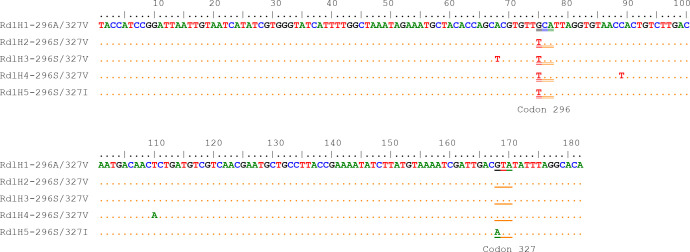
Nucleotide sequence alignment of the five *Rdl* haplotypes of the *Cx. tritaeniorhynchus* identified in this study. The codon 327 and codon 296 are indicated by single underline and double underline respectively.

From the 182-bp sequences that cover the codons 296 and 327 of the Rdl gene, five haplotypes with five nucleotide polymorphic sites in total were identified (**Figure 4**). The nucleotide variation at nucleotides 75 (T to G) and 168 (G to A) resulted in amino acid changes from Ala to Ser (A296S) and from Val to Ile (V327I), respectively. Other three nucleotide differences were synonymous. Among the five phased haplotypes, one was the wild (RdlH1-AV), three were single-site (296S) mutant, and the RdlH5-SI carried two mutations (196S+327I).

All the possible genotypes for the resistance-related Rdl residue at position 296 (Rdl-296) were detected in our samples (**Table 2**). Except for YX (*P*=0.016), the Rdl A296S mutation in other five populations were within HWE (*P*>0.05). Significant differences in genotype frequencies were observed among populations (*P* < 1.19e-07). The frequency of resistant homozygote and that of resistance allele (296S) ranged from 44.44% (ST) to 90.00% (AZ), and from 62.04% (ST) to 94.00% (AZ) respectively (**Table 2**; **Figure 3B**). Inter-population comparison showed that the frequency of the A296S mutation in ST was significantly lower than that in the other 5 populations (*P*<0.01).

**Table 2:** Frequency of individual genotypes of the *Rdl* gene at position 296 in six *Cx. tritaeniorhynchus* populations in Mianyang City, Sichuan Province of China.

Locations	N	Genotypes		
		GCA	GCA/TCA	TCA
AZ	50	1 (2.00%)	4 (8.00%)	45 (90.00%)
BC	55	2 (3.60%)	9 (16.40%)	44 (80.00%)
JY	52	3 (5.77%)	11 (21.15%)	38 (73.08%)
PW	51	3 (5.88%)	11 (21.57%)	37 (72.55%)
ST	54	11 (20.37%)	19 (35.19%)	24 (44.44%)
YX*	50	5 (10.00%)	7 (14.00%)	38 (76.00%)
Total	312	25 (8.01%)	61 (19.55%)	226 (72.44%)

*Deviation from HWE.

**Figure 5:**
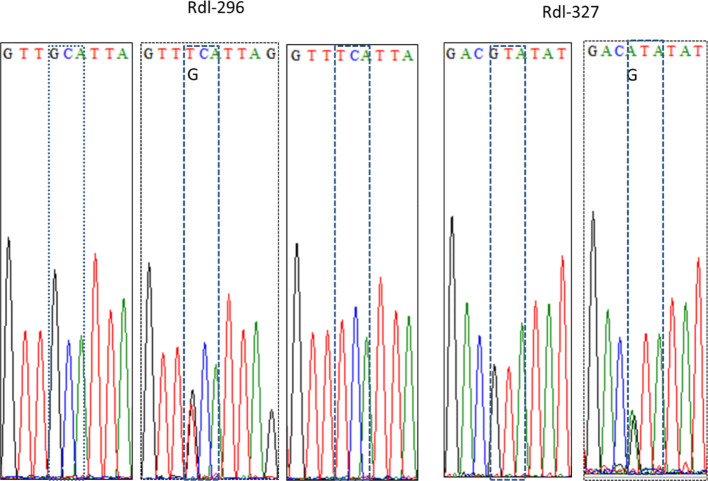
Representative DNA sequencing chromatogram of partial *Rdl* gene of the *Cx. tritaeniorhynchus*. The codons encoding the amino acid residues at position 296 and 327 are dash-boxed.

Focusing on the amino acid residue of Rdl at position 327 (Rdl-327), the wild homozygote (GTA) and heterozygote (GTA/ATA) were detected, while no resistant homozygote was observed in our samples (**Figure 5**; **Table 3**). Most individuals (> 95%) were wild homozygotes, and the frequency of the resistant allele (327I) was less than 2% (**Figure 3C**). No significant difference in the frequency of the Rdl V327I mutation was detected among populations.

**Table 3:** Frequency of individual genotypes of the *Rdl* gene at position 327 in six *Cx. tritaeniorhynchus* populations in Mianyang City, Sichuan Province of China.

Locations	N	Genotypes		
		GTA	GTA/ATA	ATA
AZ	50	49 (98.00%)	1 (2.00%)	0
BC	55	53 (96.40%)	2 (3.60%)	0
JY	52	51 (98.08%)	1 (1.92%)	0
PW	52	51 (98.08%)	1 (1.92%)	0
ST	54	53 (98.15%)	1 (1.85%)	0
YX	50	49 (98.00%)	1 (2.00%)	0
Total	313	306 (97.76%)	7 (22.36%)	0

### 3.3 Distribution and frequency of the AChE F455W mutation

**Figure 6:**
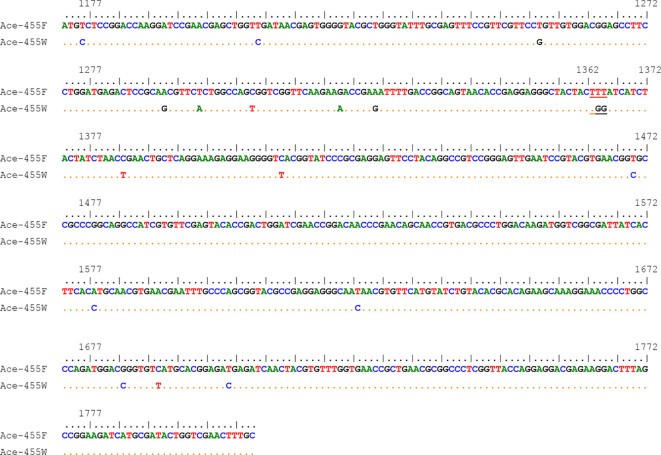
Nucleotide sequence alignment of the two *Ace* haplotypes of the *Cx. tritaeniorhync*hus identified in this study. The codon 455 is indicated by single underline. The numbers above the sequences are corresponding to the positions of the coding sequence (GenBank accession No. AB122152.1).

A total of 18 nucleotide polymorphic sites were observed in the 633-bp fragments of the *Ace* gene (**Figure 6**). Except for nucleotides 1364 and 1365 that lead to an amino acid substitution, other variations were synonymous. Two haplotypes (the wild *Ace*-455F and the resistant *Ace*-455W) were identified from our samples (**Figure 6**).

The frequency of resistant (*Ace*-455W) homozygote was above 96% in all populations (**Table 4**). The presence of wild homozygote was at a very low frequency (1.96%), and only detected in JY. *Ace-*455F/W heterozygotes were distributed in 4 out of the 6 populations, but its frequency was very low (< 5%) (**Figure 3D**). Notably, the frequency of resistance allele (*Ace*-455W) was more than 97%, and the 455W mutation was fixed in AZ and YX populations (**Figure 3D**). Overall, there was no significant difference in the frequency of the AChE F455W mutation among different populations (*P*= 0.381230).

**Table 4:** Frequency of individual genotypes of the *Ace* gene at position 455 in six *Cx. tritaeniorhynchus* populations in Mianyang City, Sichuan Province of China.

Locations	N	Genotypes		
		TTT	TTT/TGG	TGG
AZ	52	0	0	52 (100.00%)
BC	55	0	2 (3.60%)	53 (96.40%)
JY*	51	1 (1.96%)	1 (1.96%)	49 (96.08%)
PW	53	0	1 (1.89%)	52 (98.11%)
ST	51	0	2 (3.92%)	49 (96.08%)
YX	48	0	0	48 (100.00%)
Total	310	1 (0.32%)	6 (1.94%)	303 (97.74%)

*Deviation from HWE.

### 3.4 Distribution and frequency of triple−locus genotype combinations

From the examined samples, 14 triple-locus genotype combinations were documented (**Table 5**). Type A (LL-SS-WW) and B (LF-SS-WW) were the two dominant combinations, which were distributed across Mianyang City. Notably, both Type A and Type B were resistant homozygotes for both *Rdl* and *Ace*. In addition, there were resistant individuals homozygous for the three target genes (Type N) in four sites (AZ,JY,ST,YX) despite low frequencies.

**Table 5:** Distribution and frequency (%) of triple-locus genotype combinations in six *Cx. tritaeniorhynchus* populations in Mianyang City, Sichuan Province of China.

Type	VGSC1014-Rdl296-Ace455	AZ	BC	JY	PW	ST	YX
A	LL-SS-WW	60	45.5	49	58.9	23.4	48.9
B	LF-SS-WW	30	34.5	19.6	15.7	19.1	25.5
C	LL-AS-WW	6	9.1	7.8	13.7	8.5	12.8
D	LF-AS-WW	2	3.6	9.8	3.9	17.0	2.1
E	LL-AS-FW	0	3.6	0	0	0	0
F	LL-AA-WW	0	1.8	5.9	0	12.8	2.1
G	LF-AA -WW	0	1.8	0	1.96	8.5	2.1
H	LL-SS-FW	0	0	1.9	0	0	0
I	LL-AS-FF	0	0	1.9	0	0	0
J	LF-AS -FW	0	0	0	1.96	2.1	0
K	LF-SS-FW	0	0	0	0	2.1	0
L	FF-AA-WW	0	0	0	11.96		4.3
M	FF-AS-WW	0	0	0	1.96	4.3	0
N	FF-SS-WW	2	0	3.9	0	2.1	2.1

The resistant mutations are indicated in red characters.

## 4 Discussion

Although other measures help, chemical insecticides have long been used to prevent and control mosquito biting and mosquito-borne diseases. Consequently, many mosquito populations have developed resistance to commonly used insecticides (Pai et al., 2023; Tungu et al., 2023). Considering the facts that *Cx. tritaeniorhynchus* is the dominant mosquito species, and little is known about the status and involved mechanism of insecticide resistance in *Cx. tritaeniorhynchus* populations in Sichuan Province including Mianyang City, we investigated the occurrence of insecticide resistance-related genetic mutations.

Previous studies have well demonstrated that the L1014F mutation in VGSC is commonly present in several species of mosquitoes including *Cx. tritaeniorhynchus* (Liu, 2015; Wu et al., 2016; Liu et al., 2023). As expected, this mutation was also detected in *Cx. tritaeniorhynchus* samples collected from Mianyang City. The frequency of resistant allele (1014F) ranged from 16.98% (PW) to 27.78% (ST), which is similar to that reported in *Cx. tritaeniorhynchus* samples in Neijiang City of Sichuan Province (Liu et al., 2023), and in 12 sites in other four provinces of China (Wu et al., 2016).

The point mutation resulting in a substitution of Ala 296 to Ser (A296S) in the channel-lining region within the Rdl molecule has been documented for dieldrin resistance in several species of mosquitoes (Liu, 2015). In Sichuan, the A296S mutation is present at high frequencies in many populations of *Anopheles sinensis* (Qian et al., 2021). In this study, we found that the conserved A296S mutation was distributed in *Cx. tritaeniorhynchus* populations in Mianyang. To our knowledge, this is the first record of Rdl A296S mutation in this mosquito species. Notably, The A296S mutation was present at high frequencies (62.04% to 94.00%). In addition to the V327I mutation which has been reported to coexist with A296S in dieldrin-resistant *An. funestus* (Wondji et al., 2011) and in *An. sinensis* (Qian et al., 2021), a low frequency (0.93% to 1.80%) of the same V327I mutation was detected for the first time in *Cx. tritaeniorhynchus* in this study. Interestingly, the V327I was found only in individuals that harbored the A296S mutation. Until now, the impact of the V327I mutation on Rdl sensitivity remains unknown and is worthy of further investigation.

The Gly-119-Ser (G119S) substitution in AChE is commonly detected in mosquitoes (Liu, 2015), but not reported in *Cx. tritaeniorhynchus*. Instead, the F455W mutation leading to insensitivity of AChE to OP and CB has been identified in *Cx. tritaeniorhynchu*s from Japan (Nabeshima et al., 2004), India (Misra and Gore, 2015), and China (Wu et al., 2016; Liu et al., 2023). The distribution scenario of the AChE F455W mutation observed in this study further supports the notion that the resistance *Ace*-455W allele is widely distributed at a very high frequency in China (Wu et al., 2016; Liu et al., 2023).

## 5 Conclusions

The results revealed the prevalent existence of resistance-conferring mutations in multiple insecticide target proteins of *Cx. tritaeniorhynchus* in Mianyang City. The co-occurrence of Ace- F455W and Rdl-296S at high levels warns a risk of failure in the control of *Cx. tritaeniorhynchus* using chemical insecticides targeting the AChE and Rdl in this region. It is highly recommended to monitor phenotypic resistance in *Cx. tritaeniorhynchus* on a regular basis.

## Data Availability

The original contributions presented in the study are included in the article/supplementary material. Further inquiries can be directed to the corresponding author.
